# Bis(7-amino-1,2,4-triazolo[1,5-*a*]pyrimidin-4-ium) bis­(oxalato-κ^2^ *O* ^1^,*O* ^2^)cuprate(II) dihydrate

**DOI:** 10.1107/S1600536811040724

**Published:** 2011-10-12

**Authors:** Ana B. Caballero, Óscar Castillo, Antonio Rodríguez-Diéguez, Juan M. Salas

**Affiliations:** a Departamento de Química Inorgánica, Facultad de Ciencias, Universidad de Granada, c/ Severo Ochoa s/n, E-18071 Granada, Spain; b Departamento de Química Inorgánica, Facultad de Ciencia y Tecnología, Universidad del País Vasco, Apdo. 644, E-48080 Bilbao, Spain

## Abstract

The structure of the title ionic compound, (C_5_H_6_N_5_)_2_[Cu(C_2_O_4_)_2_]·2H_2_O, consists of a centrosymmetric copper(II) oxalate dianion, two monoprotonated mol­ecules of the adenine analog 7-amino-1,2,4-triazolo[1,5-*a*]pyrimidine (7atp) and two water mol­ecules of crystallization. The Cu^II^ ion, located on an inversion center, exhibits a sligthly distorted square-planar coordination geometry, in which two oxalate anions bind in a bidentate fashion. The triazolopyrimidine ligand is protonated at the N atom in position 4, instead of its most basic N atom in position 3. This fact may be explained by the network stability, which is provided through the formation of a two-dimensional wave-like network parallel to (50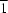) by N—H⋯O, O—H⋯N and O—H⋯O hydrogen bonds. These nets are further connected *via* C—H⋯O inter­actions.

## Related literature

For the design and synthesis of biomimetic systems, see: Hannon (2007 ▶); Legraverend & Grierson (2006 ▶). For the coordination chemistry of 1,2,4-triazolo[1,5-*a*]pyrimidine derivatives, see: Salas *et al.* (1999 ▶); Caballero *et al.* (2011 ▶). For coordination compounds of the protonated form of triazolopyrimidine, most of them bearing the 5,7-dimethyl­ated derivative, see: Szlyk *et al.* (2002 ▶); Maldonado *et al.* (2005 ▶, 2008 ▶). 
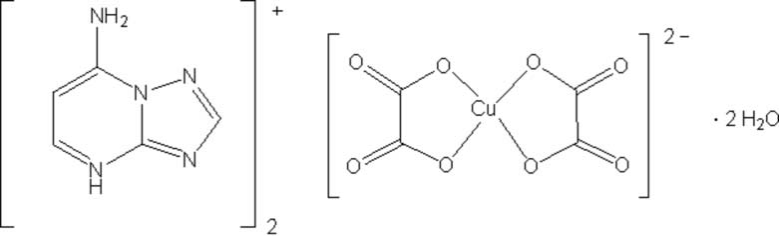


## Experimental

### 

#### Crystal data

(C_5_H_6_N_5_)_2_[Cu(C_2_O_4_)_2_]·2H_2_O
*M* *_r_* = 547.91
Monoclinic, 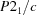

*a* = 3.6599 (2) Å
*b* = 24.1977 (10) Å
*c* = 11.1963 (5) Å
β = 92.344 (4)°
*V* = 990.73 (8) Å^3^
*Z* = 2
Mo *K*α radiation
μ = 1.19 mm^−1^
*T* = 293 K
0.59 × 0.07 × 0.05 mm

#### Data collection

Oxford Diffraction Xcalibur CCD diffractometer
Absorption correction: analytical (*CrysAlis RED*; Oxford Diffraction, 2006 ▶) *T*_min_ = 0.675, *T*_max_ = 0.950
8945 measured reflections
2170 independent reflections
1476 reflections with *I* > 2σ(*I*)
*R*_int_ = 0.039

#### Refinement

*R*[*F* ^2^ > 2σ(*F* ^2^)] = 0.037
*wR*(*F* ^2^) = 0.092
*S* = 0.91
2170 reflections
160 parameters
H-atom parameters constrained
Δρ_max_ = ? e Å^−3^
Δρ_min_ = ? e Å^−3^

### 

Data collection: *CrysAlis CCD* (Oxford Diffraction, 2006 ▶); cell refinement: *CrysAlis CCD*; data reduction: *CrysAlis RED* (Oxford Diffraction, 2006 ▶); program(s) used to solve structure: *SIR92* (Altomare *et al.*, 1994 ▶); program(s) used to refine structure: *SHELXL97* (Sheldrick, 2008 ▶); molecular graphics: *ORTEP-3* (Farrugia, 1997 ▶); software used to prepare material for publication: *WinGX* (Farrugia, 1999 ▶).

## Supplementary Material

Crystal structure: contains datablock(s) global, I. DOI: 10.1107/S1600536811040724/su2318sup1.cif

Structure factors: contains datablock(s) I. DOI: 10.1107/S1600536811040724/su2318Isup2.hkl

Additional supplementary materials:  crystallographic information; 3D view; checkCIF report

## Figures and Tables

**Table 1:** Hydrogen-bond geometry (Å, °)

*D*—H⋯*A*	*D*—H	H⋯*A*	*D*⋯*A*	*D*—H⋯*A*
N4*A*—H4*A*⋯O3^i^	0.86	1.85	2.685 (3)	165
O1*W*—H11*W*⋯N1*A*^ii^	0.86	2.38	3.197 (4)	157
O1*W*—H12*W*⋯O1^iii^	0.86	2.15	2.985 (4)	163
N7*A*—H71*A*⋯O4^iv^	0.86	2.00	2.856 (3)	170
N7*A*—H72*A*⋯O1*W*	0.86	2.01	2.793 (4)	151
C2*A*—H2*A*⋯O2^v^	0.93	2.46	3.320 (3)	154
C5*A*—H5*A*⋯O2^i^	0.93	2.45	3.126 (3)	129
C6*A*—H6*A*⋯O3^iv^	0.93	2.39	3.246 (3)	153

Symmetry codes: (i) 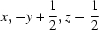; (ii) 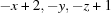; (iii) 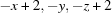; (iv) 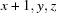; (v) 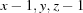.

**Fig. 1.**
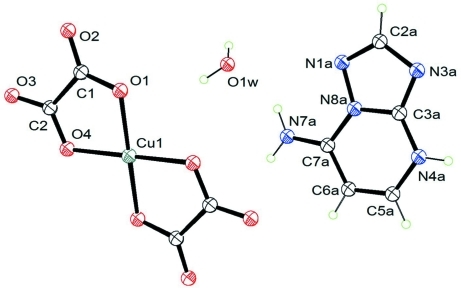
ORTEP representation of the asymmetric unit of the title compound, showing the atom labels and displacement ellipsoids drawn at the 50% probability level.

**Fig. 2.**
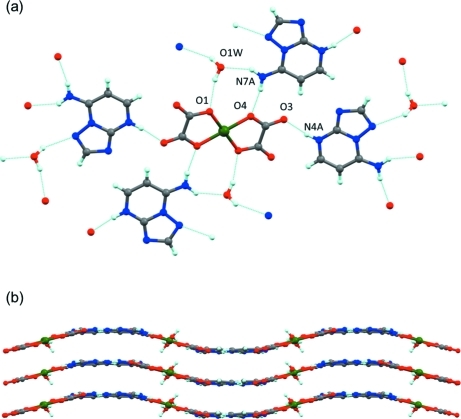
View along the a-axis showing the formation of the hydrogen bonded (thin blue lines) layer (a); view of the packing of the layers (b).

## supplementary crystallographic information

### Comment

In the recent years, the rational design and synthesis of biomimetic systems based on the interaction of biologically relevant molecules with inorganic species has aroused a remarkable research interest. The study of these systems not only stems from the desire to better understand the complex interactions often present in different molecular biorecognition processes (Hannon, 2007), but also to afford a powerful tool for the improvement of pharmaceutical agents (Legraverend & Grierson, 2006). 1,2,4-triazolo[1,5-*a*]pyrimidines may be used as purine analogs to obtain new biomimetic systems with interesting physical and biological properties, which might differ from those of purine-based systems due to their slightly different atomic arrangement. Previous studies revealed that the coordination chemistry of 1,2,4-triazolo[1,5-*a*]pyrimidine derivatives displays great versatility, binding metal ions in several different ways, either in a monodentate (usually through the N atom in position 3), or in a bidentate fashion, bridging metal atoms and leading to binuclear or polynuclear species with interesting metal-metal interactions (Salas *et al.*, 1999; Caballero *et al.*, 2011). However, few examples containing a protonated form of the triazolopyrimidine have been reported, most of them bearing the 5,7-dimethylated derivative (Szlyk *et al.*, 2002; Maldonado *et al.*, 2005; Maldonado *et al.*, 2008). To the best of our knowledge, the copper(II) complex reported herein is the only one obtained with the protonated form of the 7-amino derivative, 7-amino-1,2,4-triazolo[1,5-*a*]pyrimidine (7atp).

The molecular structure of the title compound is illustrated in Fig. 1. It consists of an dianionic copper(II) oxalate complex, showing a square planar geometry, two monoprotonated 7atp cations and two crystallization water molecules. The copper(II) ion is coordinated to two oxalate anions, which display a bidentate \k^2^O,*O*' mode and are related by an inversion centre. The protonation of 7atp occured through the N atom in position 4, N4, in a neutral-slightly basic media. This may be favoured by the formation of a stable two-dimensional network built from N-H···O, O-H···N and O-H···O hydrogen bonds involving the crystallization water molecules, the oxalate O atoms and the amine group (Table 1 and Fig. 2). These nets are further connected via C-H···O interactions (Table 1).

### Experimental

The title compound was prepare by dissolving K_2_[Cu(ox)_2_].2H_2_O (0.16 mmol, 0.057 g) in 10 ml of a hot aqueous solution of potassium oxalate dihydrated (0.16 mmol, 0.029 g). 10 ml of an aqueous solution of 7-amino-1,2,4-triazolo[1,5-*a*]pyrimidine (7atp) (0.032 mmol, 0.043 g) was then added and the colour changed from blue to green. The solution was stirred at 50° C for 30 min, and then left standing at room temperature. After one day, prismatic dark-green crystals of the polymeric complex {[Cu(ox)(7atp)_2_].3H_2_O}_n_ were formed and filtered off. After 3–4 days needle-shaped blue crystals of the title complex were isolated and used for the present X-ray diffraction studies.

### Refinement

The H atoms were positioned geometrically and treated as riding atoms: O-H = 0.86 Å, N-H = 0.86 Å, C—H = 0.93 Å, with *U*_iso_(H) = k × *U*_eq_(O,N,C) where k = 1.5 for the water H atoms, and k = 1.2 for all other H atoms.

### Crystal data

**Table:**

(C_5_H_6_N_5_)_2_[Cu(C_2_O_4_)_2_]·2H_2_O	*F*(000) = 558
*M**_r_* = 547.91	*D*_x_ = 1.837 Mg m^−^^3^
Monoclinic, *P*2_1_/*c*	Mo *K*α radiation, λ = 0.71073 Å
Hall symbol: -P 2ybc	Cell parameters from 8945 reflections
*a* = 3.6599 (2) Å	θ = 3.1–27.1°
*b* = 24.1977 (10) Å	µ = 1.19 mm^−^^1^
*c* = 11.1963 (5) Å	*T* = 293 K
β = 92.344 (4)°	Needle, blue
*V* = 990.73 (8) Å^3^	0.59 × 0.07 × 0.05 mm
*Z* = 2	

### Data collection

**Table:**

Oxford Diffraction Xcalibur CCD diffractometer	2170 independent reflections
Radiation source: fine-focus sealed tube	1476 reflections with *I* > 2σ(*I*)
graphite	*R*_int_ = 0.039
ω scans	θ_max_ = 27.1°, θ_min_ = 3.1°
Absorption correction: analytical (*CrysAlis RED*; Oxford Diffraction, 2006)	*h* = −3→4
*T*_min_ = 0.675, *T*_max_ = 0.950	*k* = −30→30
8945 measured reflections	*l* = −14→13

### Refinement

**Table:**

Refinement on *F*^2^	Primary atom site location: structure-invariant direct methods
Least-squares matrix: full	Secondary atom site location: difference Fourier map
*R*[*F*^2^ > 2σ(*F*^2^)] = 0.037	Hydrogen site location: inferred from neighbouring sites
*wR*(*F*^2^) = 0.092	H-atom parameters constrained
*S* = 0.91	*w* = 1/[σ^2^(*F*_o_^2^) + (0.0552*P*)^2^] where *P* = (*F*_o_^2^ + 2*F*_c_^2^)/3
2170 reflections	(Δ/σ)_max_ < 0.001
160 parameters	Δρ_max_ = 0.47 e Å^−^^3^
0 restraints	Δρ_min_ = −0.42 e Å^−^^3^

### Special details

**Table:**

Experimental. (CrysAlis RED; Oxford Diffraction, 2006) Analytical numeric absorption correction using a multifaceted crystal.
Geometry. Bond distances, angles etc. have been calculated using the rounded fractional coordinates. All su's are estimated from the variances of the (full) variance-covariance matrix. The cell esds are taken into account in the estimation of distances, angles and torsion angles
Refinement. Refinement of *F*^2^ against ALL reflections. The weighted *R*-factor *wR* and goodness of fit *S* are based on *F*^2^, conventional *R*-factors *R* are based on *F*, with *F* set to zero for negative *F*^2^. The threshold expression of *F*^2^ > σ(*F*^2^) is used only for calculating *R*-factors(gt) *etc*. and is not relevant to the choice of reflections for refinement. *R*-factors based on *F*^2^ are statistically about twice as large as those based on *F*, and *R*- factors based on ALL data will be even larger.

### Fractional atomic coordinates and isotropic or equivalent isotropic displacement parameters (Å^2^)

**Table:**

	*x*	*y*	*z*	*U*_iso_*/*U*_eq_	
Cu1	0.50000	0.00000	1.00000	0.0235 (2)	
O1	0.8421 (5)	0.03719 (7)	1.10908 (16)	0.0295 (6)	
O2	1.0514 (6)	0.12235 (8)	1.14517 (17)	0.0308 (7)	
O3	0.6886 (6)	0.15536 (7)	0.93468 (16)	0.0305 (7)	
O4	0.4741 (5)	0.06976 (7)	0.91704 (15)	0.0238 (6)	
C1	0.8723 (7)	0.08907 (11)	1.0863 (2)	0.0203 (8)	
C2	0.6629 (8)	0.10777 (10)	0.9703 (2)	0.0209 (8)	
N1A	0.7487 (7)	0.11549 (9)	0.4729 (2)	0.0255 (7)	
N3A	0.6315 (6)	0.20038 (10)	0.39005 (19)	0.0259 (7)	
N4A	0.9285 (6)	0.25338 (9)	0.5496 (2)	0.0230 (7)	
N7A	1.1147 (7)	0.10032 (9)	0.6956 (2)	0.0297 (8)	
N8A	0.8882 (6)	0.15695 (8)	0.54508 (18)	0.0193 (7)	
C2A	0.6026 (8)	0.14399 (12)	0.3835 (2)	0.0264 (9)	
C3A	0.8143 (8)	0.20707 (10)	0.4929 (2)	0.0203 (8)	
C5A	1.1084 (8)	0.24886 (11)	0.6569 (2)	0.0250 (9)	
C6A	1.1821 (8)	0.19969 (11)	0.7099 (2)	0.0238 (8)	
C7A	1.0687 (7)	0.15005 (11)	0.6543 (2)	0.0215 (8)	
O1W	0.9004 (13)	−0.00801 (11)	0.6425 (3)	0.1037 (16)	
H2A	0.48460	0.12670	0.31850	0.0320*	
H4A	0.88760	0.28530	0.51800	0.0280*	
H5A	1.18500	0.28100	0.69600	0.0300*	
H6A	1.30870	0.19870	0.78360	0.0290*	
H71A	1.22680	0.09520	0.76370	0.0360*	
H72A	1.03300	0.07250	0.65480	0.0360*	
H11W	1.00140	−0.03050	0.59390	0.1560*	
H12W	0.95310	−0.02300	0.71050	0.1560*	

### Atomic displacement parameters (Å^2^)

**Table:**

	*U*^11^	*U*^22^	*U*^33^	*U*^12^	*U*^13^	*U*^23^
Cu1	0.0302 (3)	0.0162 (2)	0.0232 (3)	−0.0033 (2)	−0.0109 (2)	0.0037 (2)
O1	0.0395 (13)	0.0194 (9)	0.0280 (11)	−0.0060 (9)	−0.0163 (9)	0.0058 (8)
O2	0.0368 (13)	0.0263 (10)	0.0282 (11)	−0.0057 (9)	−0.0135 (9)	−0.0037 (9)
O3	0.0431 (13)	0.0175 (10)	0.0297 (11)	−0.0070 (9)	−0.0121 (9)	0.0062 (8)
O4	0.0318 (12)	0.0176 (9)	0.0210 (10)	−0.0046 (8)	−0.0126 (8)	0.0031 (7)
C1	0.0203 (15)	0.0206 (13)	0.0199 (13)	−0.0003 (12)	−0.0013 (11)	0.0007 (11)
C2	0.0231 (15)	0.0197 (13)	0.0198 (14)	−0.0013 (12)	0.0001 (11)	−0.0015 (11)
N1A	0.0287 (14)	0.0247 (12)	0.0226 (12)	−0.0018 (11)	−0.0032 (10)	−0.0034 (9)
N3A	0.0259 (13)	0.0319 (13)	0.0197 (12)	0.0030 (11)	−0.0022 (10)	0.0052 (10)
N4A	0.0253 (13)	0.0185 (11)	0.0248 (12)	0.0040 (10)	−0.0019 (10)	0.0034 (9)
N7A	0.0424 (16)	0.0253 (13)	0.0203 (12)	0.0031 (12)	−0.0109 (11)	0.0036 (10)
N8A	0.0216 (13)	0.0206 (11)	0.0156 (11)	0.0012 (10)	−0.0020 (9)	0.0007 (9)
C2A	0.0243 (16)	0.0347 (16)	0.0199 (15)	−0.0011 (14)	−0.0026 (12)	−0.0033 (12)
C3A	0.0184 (14)	0.0221 (13)	0.0205 (14)	0.0037 (12)	0.0017 (11)	0.0038 (11)
C5A	0.0215 (15)	0.0282 (15)	0.0253 (15)	0.0000 (12)	−0.0003 (12)	−0.0058 (12)
C6A	0.0211 (15)	0.0311 (15)	0.0188 (14)	0.0003 (13)	−0.0049 (11)	0.0006 (12)
C7A	0.0190 (15)	0.0282 (14)	0.0174 (13)	0.0018 (12)	0.0021 (11)	0.0036 (11)
O1W	0.196 (4)	0.0385 (16)	0.075 (2)	0.000 (2)	−0.014 (2)	−0.0046 (14)

### Geometric parameters (Å, °)

**Table:**

Cu1—O1	1.9349 (18)	N3A—C2A	1.370 (4)
Cu1—O4	1.9272 (17)	N4A—C5A	1.351 (3)
Cu1—O1^i^	2.8879 (18)	N4A—C3A	1.346 (3)
Cu1—O1^ii^	1.9349 (18)	N7A—C7A	1.298 (3)
Cu1—O4^ii^	1.9272 (17)	N8A—C3A	1.368 (3)
Cu1—O1^iii^	2.8879 (18)	N8A—C7A	1.376 (3)
O1—C1	1.287 (3)	N4A—H4A	0.8600
O2—C1	1.215 (3)	N7A—H72A	0.8600
O3—C2	1.224 (3)	N7A—H71A	0.8600
O4—C2	1.283 (3)	C1—C2	1.548 (3)
O1W—H11W	0.8600	C5A—C6A	1.352 (4)
O1W—H12W	0.8600	C6A—C7A	1.408 (4)
N1A—C2A	1.311 (3)	C2A—H2A	0.9300
N1A—N8A	1.374 (3)	C5A—H5A	0.9300
N3A—C3A	1.318 (3)	C6A—H6A	0.9300
			
O1—Cu1—O4	85.10 (7)	C3A—N4A—H4A	121.00
O1—Cu1—O1^i^	96.74 (6)	C5A—N4A—H4A	121.00
O1—Cu1—O1^ii^	180.00	H71A—N7A—H72A	120.00
O1—Cu1—O4^ii^	94.90 (7)	C7A—N7A—H72A	120.00
O1—Cu1—O1^iii^	83.26 (6)	C7A—N7A—H71A	120.00
O1^i^—Cu1—O4	84.58 (6)	O1—C1—O2	126.0 (2)
O1^ii^—Cu1—O4	94.90 (7)	O2—C1—C2	119.9 (2)
O4—Cu1—O4^ii^	180.00	O1—C1—C2	114.1 (2)
O1^iii^—Cu1—O4	95.43 (6)	O3—C2—C1	120.4 (2)
O1^i^—Cu1—O1^ii^	83.26 (6)	O4—C2—C1	114.8 (2)
O1^i^—Cu1—O4^ii^	95.43 (6)	O3—C2—O4	124.8 (2)
O1^i^—Cu1—O1^iii^	180.00	N1A—C2A—N3A	117.0 (2)
O1^ii^—Cu1—O4^ii^	85.10 (7)	N3A—C3A—N4A	130.6 (2)
O1^ii^—Cu1—O1^iii^	96.74 (6)	N4A—C3A—N8A	119.0 (2)
O1^iii^—Cu1—O4^ii^	84.58 (6)	N3A—C3A—N8A	110.4 (2)
Cu1—O1—C1	112.79 (15)	N4A—C5A—C6A	122.9 (2)
Cu1—O1—Cu1^iv^	96.74 (7)	C5A—C6A—C7A	120.5 (2)
Cu1^iv^—O1—C1	98.07 (15)	N8A—C7A—C6A	114.3 (2)
Cu1—O4—C2	112.90 (15)	N7A—C7A—C6A	127.0 (2)
H11W—O1W—H12W	102.00	N7A—C7A—N8A	118.7 (2)
N8A—N1A—C2A	101.3 (2)	N3A—C2A—H2A	121.00
C2A—N3A—C3A	101.8 (2)	N1A—C2A—H2A	121.00
C3A—N4A—C5A	118.9 (2)	N4A—C5A—H5A	119.00
C3A—N8A—C7A	124.5 (2)	C6A—C5A—H5A	119.00
N1A—N8A—C3A	109.5 (2)	C5A—C6A—H6A	120.00
N1A—N8A—C7A	126.0 (2)	C7A—C6A—H6A	120.00
			
O4—Cu1—O1—C1	5.64 (17)	C2A—N3A—C3A—N8A	−0.3 (3)
O4—Cu1—O1—Cu1^iv^	−96.07 (7)	C5A—N4A—C3A—N3A	178.9 (3)
O1^i^—Cu1—O1—C1	−78.29 (17)	C3A—N4A—C5A—C6A	0.5 (4)
O4^ii^—Cu1—O1—C1	−174.36 (17)	C5A—N4A—C3A—N8A	−0.8 (4)
O1^iii^—Cu1—O1—C1	101.71 (17)	C7A—N8A—C3A—N4A	1.2 (4)
O1—Cu1—O4—C2	−3.55 (18)	C3A—N8A—C7A—C6A	−1.1 (4)
O1^i^—Cu1—O4—C2	93.71 (18)	C7A—N8A—C3A—N3A	−178.6 (2)
O1^ii^—Cu1—O4—C2	176.45 (18)	N1A—N8A—C3A—N3A	0.3 (3)
O1^iii^—Cu1—O4—C2	−86.29 (18)	N1A—N8A—C3A—N4A	180.0 (2)
Cu1—O1—C1—O2	175.4 (2)	N1A—N8A—C7A—C6A	−179.7 (2)
Cu1—O1—C1—C2	−6.3 (3)	N1A—N8A—C7A—N7A	−0.3 (4)
Cu1^iv^—O1—C1—O2	−83.8 (3)	C3A—N8A—C7A—N7A	178.4 (3)
Cu1^iv^—O1—C1—C2	94.6 (2)	O2—C1—C2—O4	−178.0 (2)
Cu1—O4—C2—O3	180.0 (2)	O2—C1—C2—O3	3.1 (4)
Cu1—O4—C2—C1	1.1 (3)	O1—C1—C2—O3	−175.4 (2)
N8A—N1A—C2A—N3A	−0.2 (3)	O1—C1—C2—O4	3.6 (3)
C2A—N1A—N8A—C3A	0.0 (3)	N4A—C5A—C6A—C7A	−0.5 (4)
C2A—N1A—N8A—C7A	178.8 (2)	C5A—C6A—C7A—N7A	−178.7 (3)
C3A—N3A—C2A—N1A	0.4 (3)	C5A—C6A—C7A—N8A	0.8 (4)
C2A—N3A—C3A—N4A	180.0 (3)		

Symmetry codes: (i) *x*−1, *y*, *z*; (ii) −*x*+1, −*y*, −*z*+2; (iii) −*x*+2, −*y*, −*z*+2; (iv) *x*+1, *y*, *z*.

### Hydrogen-bond geometry (Å, °)

**Table:**

*D*—H···*A*	*D*—H	H···*A*	*D*···*A*	*D*—H···*A*
N4A—H4A···O3^v^	0.86	1.85	2.685 (3)	165
O1W—H11W···N1A^vi^	0.86	2.38	3.197 (4)	157
O1W—H12W···O1^iii^	0.86	2.15	2.985 (4)	163
N7A—H71A···O4^iv^	0.86	2.00	2.856 (3)	170
N7A—H72A···O1W	0.86	2.01	2.793 (4)	151
N7A—H72A···N1A	0.86	2.48	2.806 (3)	103
C2A—H2A···O2^vii^	0.93	2.46	3.320 (3)	154
C5A—H5A···O2^v^	0.93	2.45	3.126 (3)	129
C6A—H6A···O3^iv^	0.93	2.39	3.246 (3)	153

Symmetry codes: (v) *x*, −*y*+1/2, *z*−1/2; (vi) −*x*+2, −*y*, −*z*+1; (iii) −*x*+2, −*y*, −*z*+2; (iv) *x*+1, *y*, *z*; (vii) *x*−1, *y*, *z*−1.
